# EXPANDING CDC’S STILL GOING STRONG CAMPAIGN TO INCLUDE TRIBAL POPULATIONS AND PROMOTE SOCIAL CONNECTEDNESS

**DOI:** 10.1093/geroni/igad104.3323

**Published:** 2023-12-21

**Authors:** Yara Haddad, Gwen Bergen, Erin Bruner

**Affiliations:** Centers for Disease Control and Prevention, Atlanta, Georgia, United States; Centers for Disease Control and Prevention, Atlanta, Georgia, United States; Centers for Disease Control and Prevention, Atlanta, Georgia, United States

## Abstract

The Centers for Disease Control and Prevention (CDC) launched the Still Going Strong (SGS) campaign in 2021 to promote ways older adults (65+) can age without injury. The goal was to encourage older adult participation in lifelong activities while taking steps to prevent injuries. Based on initial campaign evaluation, CDC plans to expand SGS to include social connectedness content and reflect greater diversity. To inform this expansion, CDC conducted spring 2023 focus groups with Tribal elders (n=8) and differently-abled older adults (n=9) and presented different concepts depicting older adults engaging in activities with others. Tribal elders were affiliated with five tribes: Navajo, Ojibwa, Citizen Potawatomi, Lumbee Indian, and Wailaki. Half were female (n=4), and majority were 63–70 years of age (n=5). This group tested two new radio concepts: (1) Walking and Talking and (2) Sharing Lessons. Themes across Tribal participants included the importance of connecting with loved ones, sharing culture and spirituality through service, and connecting with younger generations. The differently abled focus group was majority female (n=6), white, (n=5), 70–75 years of age (n=5) and reported using a mobility aid (n=6). This group tested two new video concepts: (1) Best Friends and (2) Health in Numbers. Participants identified social connection and being with others as important for their overall health. Using these data, we will create and refine campaign materials targeting both populations, which will be disseminated and further evaluated. By creating culturally relevant and respectful health promotion messages, we can better engage disproportionately affected persons and reduce injuries.

